# Supplementary material for: Near-infrared photoimmunotherapy is effective treatment for colorectal cancer in orthotopic nude-mouse models
Source: PLoS One. 2020 Jun 18;15(6):e0234643. doi: 10.1371/journal.pone.0234643 (PMC7302706; doi:10.1371/journal.pone.0234643)
Supplement: Attachment — Submitted filename: Review PIT for colorectal cancer.docx [file pone.0234643.s001.docx]

PIT in colorectal cancer.

This publication uses near infrared laser activation to drive tumor cell death by activation of an antibody conjugated to a dye that can be activated by near infrared light.

To evaluate this conjugate, humanized antibody to carcinoembryonic antigen was linked to the dye. Two colon cancer cell lines were injected subcutaneously into nude mice. In addition, orthotopic colon cancer models were established by surgical implantation of LS174T tumor fragments onto the cecum.

Activity was assessed by laser treatment alone with no antibody conjugate.

To perform study, 3 groups of mice were treated: Control which received laser treatment alone without the antibody conjugate. A second grade received one treatment with PIT and the third group received repeated dosing of PIT. Tumors were established and allowed to grow for 3 weeks prior to treatment. Groups 2 and 3 he received the antibody conjugate. Laser treatment was administered over 30 minutes by a standardize protocol either one time, group 2, or multiple times, group 3. A shema of the approach as shown in figure 1.

Figure 2 demonstrates a highly significant reduction in tumor viability with increasing laser intensity. Significant reduction in fluorescence activity is shown following PIT treatment in the subcutaneous flank model. Moreover, as shown in figure 5, there is effective control of the mean tumor volume and animals receiving multiple PIT treatments compared to control or only a single treatment. Imaging as demonstrated in the subcutaneous model and in the orthotopic model demonstrates activity in the treated tumors.

This study demonstrates a dose dependent tumor response with photo immunotherapy. The potential for combining tumor-specific antibodies with laser activation raises the potential for highly specific surgical treatment of tumors. The study provides important additional information, particularly with the use of the orthotopic mouse model. A critical unmet need is the persistence of tumor cells at the surgically resected margins. The use of PIT to help eradicate residual tumor at the surgical margins may help significantly reduce tumor recurrence.

Of particular interest is the potential for this approach to be used among patients with peritoneal metastases. Currently, the options for management for these patients this limited and additional options, as demonstrated in this research, are critical to the field.
