# Supplementary material for: Near-infrared photoimmunotherapy is effective treatment for colorectal cancer in orthotopic nude-mouse models
Source: PLoS One. 2020 Jun 18;15(6):e0234643. doi: 10.1371/journal.pone.0234643 (PMC7302706; doi:10.1371/journal.pone.0234643)
Supplement: Attachment — Submitted filename: Response to Reviewers_2.docx [file pone.0234643.s003.docx]

May 7, 2020

To: Drs. Joreg Heber and Irina V. Lebedeva, Editor-in Chief and Academic Editor, PLOS One

Manuscript – “Near-Infrared Photoimmunotherapy is Effective Treatment for Colorectal Cancer in Orthotopic Nude-Mouse Models”

Drs. Heber and Lebedeva,

Thank you for your email and reviewers’ comments regarding our manuscript entitled “Near-Infrared Photoimmunotherapy is Effective Treatment for Colorectal Cancer in Orthotopic Nude-Mouse Models.” Thank you also for your interest and the thoughtful comments provided by the Reviewers. For our revision submission, we are providing a revised manuscript that addresses all Reviewer comments and suggestions.

Below, please find the Reviewer’s comments and how each was addressed in the revised manuscript. The most recent Reviewer comments are italicized and our responses to each comment are below in bold.

We thank you again for the opportunity to submit this revised manuscript and look forward to your comments.

Reviewers' Response to comments:

1. The authors state that PIT suppressed tumor growth in subcutaneous colon cancer
mouse model but this conclusion is demonstrated only in a single mouse image in
Figure 3. Quantification of fluorescence intensity and tumor size measurements need
to be provided to support this claim. Figure 4 should also be accompanied with
quantification and images of tumors.

Author’s Response: Figure 4 legend (now labelled Figure 3) and the Results now include quantification of fluorescence intensity in the orthotopic models.
Please see answer to question 2 below.

*Reviewer’s Response:
How many fluorescence measurements were calculated? It would strengthen the claim if this data was shared as a graph in updated Figure 3.*

**Figure 3 has been updated to include a graph depicting the difference in mean maximum tumor fluorescence signal before and after PIT. The figure legend provides description of how many fluorescence measurements were calculated and statistical significance. Materials and methods have been revised to include image analysis and statistical analysis of tumor fluorescence.**

2. What is the purpose of performing subcutaneous transplant studies if the authors
also perform cecal transplants, which are presumably better?

Author’s Response: Data on subcutaneous models was removed from the revised manuscript.

*Reviewer’s Response:
Author’s can include other relevant experiments as supplemental figures.*

**All relevant experiments are presented in the main manuscript.**

3. Finally, they show inhibition of tumor growth in orthotopic nude mouse models with
repeated exposure to PIT one week after initial treatment. Overall the results are
promising as this is the first study of PIT in an orthotopic mouse model of colon.
However, the conclusions drawn are largely hypothetical as the tumors were grown in
athymic nude mice without a competent immune system. Although these mice have
dendritic cells, they lack T cells. Thus, this model may have little relevance to humans.
Studies in a mouse cell line or organoid transplant model or genetically engineered
model would be more relevant.

Author’s Response: A section in the discussion has been included to discuss the limitation highlighted above: “In the present study, we utilized athymic nude mice that lack T cells, which are not representative of the complex conditions of the immune system in patients. However, even in the absence of T cells, PIT was shown to be effective, suggesting that T cells are not the major factor in immunogenic cell death. Prior to translation into clinical studies, we will perform PIT on orthotopic syngeneic models, humanized or genetically engineered mice, all with an intact immune system.”

*Reviewer’s Response:
This is acceptable.*

4. The authors also failed to discuss why the tumors rapidly increased in size following
PIT cessation in comparison to control tumors.

Author’s Response: This is an important question. We can only speculate that the first PIT caused the production of growth factors, such is sometimes the case in surgery, where residual tumors grow faster. Future experiments are needed to answer this question. This is
stated in the revised manuscript.

*Reviewer’s Response:
Histology of the tumors on future studies can also help identify characteristics of the residual tumor.*

**This point was added to the revised discussion.**

5. No experiments were performed to study the possible mechanism of action of PIT in
colon cancer. Thus, these experiments add little value to the literature on PIT.

Author’s Response: The present studies are a proof-of-principle that PIT is effective on orthotopic models of colon cancer, as previous studies were only on subcutaneous colon cancer models, which are artificial. This is stated in the revised manuscript. However, general mechanisms of PIT are discussed in the revised manuscript.

*Reviewer’s Response:
The new information provided will help readers understand the possible future directions.*

6. Why did the authors select this particular cell line, and why were studies not
repeated in additional cell lines or organoid lines?

Author’s Response:
We have done previous studies showing human colon cancer LS174T cell line is
targeted well by anti-CEA antibodies (1) and would be appropriate for an orthotopic PIT
study and proof-of-principle. This is stated in the revised manuscript.

7. As the authors themselves note, the application of this technology is questionable
since the tumor will need to be exposed to the light source. One possible application
not discussed by the authors is ablation of positive margins of adenomas during
colonoscopy.

Author’s Response:
PIT can be an intra-operative procedure; therefore, illuminating an intra-abdominal
tumor is feasible. This is discussed in the revised version. The potential application for
polyp margins during endoscopy is also now included in the Discussion.

8. Why was the treatment only provided for 1-2 weeks? This time scale is extremely
short.


Author’s Response:
The limitation of the time scale is included in the Discussion. This also includes the
reason for short time period, which was tumor growth in the control group that
exceeded the allowed tumor burden. It was discussed that survival and recurrence
studies can increase clinical applicability of PIT in colorectal cancer.

9. Histology of the tumors should be provided.


Author’s Response:
As the present study was a proof-of-principle study that PIT could arrest an orthotopic
tumor, histology studies were not done. Detailed histology studies will be done in the
future. This is discussed in the revised edition.
As part of this submission, we have included a revised version of the main manuscript
file that shows changes made by highlighting, as well as a clean version of the revised
manuscript. Again, we thank the editors and reviewers for their efforts in improving our
manuscript. We would be happy to answer any further questions and/or concerns.

As part of this submission, we have included a revised version of the main manuscript file that shows changes made by highlighting, as well as a clean version of the revised manuscript. Again, we thank the editors and reviewers for their efforts in improving our manuscript. We would be happy to answer any further questions and/or concerns.

Sincerely,

Hannah Hollandsworth, MD
